# Suboccipital craniectomy with bilateral cerebellomedullary fissure dissection for resection of a ruptured tonsillar/vermian AVM occupying the roof of the fourth ventricle in a pregnant patient

**DOI:** 10.3171/2020.10.FOCVID2059

**Published:** 2021-01-01

**Authors:** Daniel M. S. Raper, Kunal P. Raygor, Caleb Rutledge, Todd B. Dubnicoff, Adib A. Abla

**Affiliations:** Department of Neurological Surgery, University of California, San Francisco, California

## Abstract

Posterior fossa arteriovenous malformations (AVMs) in pregnant patients can present unique considerations for surgical treatment, including positioning to minimize pressure on the fetus, minimization of radiation exposure, and ethical considerations regarding emergency surgery. This video outlines surgical treatment of a ruptured tonsillar/vermian AVM performed in a staged fashion after emergent suboccipital craniotomy with posterior fossa decompression in the setting of a life-threatening infratentorial hemorrhage. Later, bilateral cerebellomedullary fissure dissection, exposure and dissection of the tela choroidea and inferior medullary velum, and disconnection of arterial feeders from the posterior inferior cerebellar artery (PICA) allowed resection of this AVM occupying the roof of the fourth ventricle.

This study was approved by the UCSF Human Research Protection Program IRB no. 18-26938.

The video can be found here: https://youtu.be/rTYUGanopUE

## Transcript

**0:22 Introduction.** This case will demonstrate resection of a tonsillar AVM in a pregnant woman in this ruptured posterior fossa AVM.^1,2^

**0:28 Clinical History.** This patient presented 18 weeks pregnant with loss of consciousness, and she was GCS of 3 with nonreactive pupils.

**0:37 Preoperative Imaging.** An EVD was placed given the hydrocephalus and IVH, and her brainstem reflexes improved, as did her pupillary response. At that time, she was taken emergently for a posterior fossa decompression.

**0:49 Continuation of Clinical History.** At the time of the posterior fossa decompression, she became unstable again, and she required defibrillation prior to turning her prone. Sinus rhythm was restored. However, she then became unstable during the craniectomy, and only a craniectomy and dural opening was performed, but no evacuation of the hematoma nor resection of the AVM given her instability. The case was stopped, and she was taken to the ICU. It was not felt that she would recover at that time given the cardiac instability; however, she did improve.

**1:22 Continuation of Preoperative Imaging.** This is her CTA at the time of presentation showing the AVM in the fourth ventricle. We can see [yellow arrow at 1:27] on the coronal CTA the draining vein coursing superiorly and to the right along the superior aspect of the AVM nidus, and it is also at the anterior aspect of the AVM nidus adjacent to the middle cerebellar peduncle on the right. We can see bilateral PICA supply from the left [yellow arrow at 1:45] and the right [yellow arrow at 1:47] PICA vessels from the telovelotonsillar segments, and we can see the AVM nidus [dashed yellow circle at 1:51] sitting here in the roof of the fourth ventricle on both the coronal and the sagittal [dashed yellow circle at 1:56] CTAs. And again, finally here, we can see this draining vein [yellow arrow at 1:59] at the anterior aspect of the nidus going superiorly, and it’s on the right side. She did improve and was following commands at 1 week.

**2:09 Patient Positioning.** She was then taken; here is her positioning in stage 2. She was taken back to the OR and placed in the lateral decubitus position.^3,4^ You can see the closure from the prior approach, or stage 1. She was placed in this position to avoid pressure on the fetus in the prone position, and the case was performed—stage 2 was performed—for the purpose of resecting the AVM.

**2:29 Demonstration of Initial Exposure After Opening Dura.** Here’s the view from the microscope during the time of surgery. We are opening, and you can see that there is scar and bipolar cautery over the surface of the cerebellum at the time of the last procedure. Again, we are opening the dura. The dura had not been completely closed from the previous procedure. We are just opening more dura along the upper cervical spinal cord to allow for access and visualization of the PICA vessels. The inferior aspect is to the right of the screen; again, she is in the lateral position, and to the left of the screen is the left PICA, where we are cutting the arachnoid. We can see the left PICA underneath the cauterized tonsil.

**3:10 Demonstration of Bilateral Telovelar Approach to Devascularize the AVM Nidus.** So, this approach—because the AVM is in bilateral tonsils and has supply from bilateral telovelotonsillar segments of PICA^5,6^—will require bilateral opening of the telovelar approach on both left and right. Here is the left-sided opening of the tela choroidea of the fourth ventricle. We can see the left PICA running in that telovelotonsillar segment. The feeders to this AVM, again, are from that telovelotonsillar segment of PICA, so we will basically skeletonize both of those segments of PICA to devascularize the AVM, and also, we will come across the superior aspect of the AVM through the inferior vermis to get all the way around the AVM. So, this is coming through the inferior vermis and, again, we will carefully cauterize. Here is an AVM clip that is needed to clip a friable perforator vessel coming through the brainstem to this AVM, which does not often respond to cautery. Additional cautery along the left side of the telovelotonsillar segment of PICA, and now we are seeing more of the right side. Here is devascalarization of feeders from the right PICA now. And then we will go around this AVM on all sides. The inferior side has already been created by the space—the foramen magnum or cisterna magna—so there is no supply from the inferior aspect of the AVM. Here we can see the draining vein [yellow arrow at 4:42] at the superior and right anterior aspect of the AVM nidus, and it will be the last pedicle of attachment of this AVM before it is removed. And we will continue to cauterize and skeletonize now the right PICA. And this is exposure of the telovelar approach on the right. And we will cauterize any remaining feeders coming through the middle cerebellar peduncle, which is seen just deep to the bipolar there (5:13)—the inferior, posterior aspect of the middle cerebellar peduncle. Again, telovelar exposure on the right. This is bipolar cautery and cutting of the last attachment of this AVM, and then it is removed.

**5:28 Demonstration of Relevant Anatomy After AVM Resection.** And now that the AVM has been removed, we can see at the inferior aspect of the screen here [yellow arrow at 5:33], we can see the upper cervical spinal cord. We can see the dura open over the cervical spine [yellow arrow at 5:38]. This is at the level of the foramen magnum [dashed yellow line at 5:41]; we can see the PICAs coming up into their telovelotonsillar segment [yellow arrow at 5:44] in the cerebellomedullary fissure. Both tonsils [shaded yellow ovals at 5:48] have been resected, and what happens when both tonsils involve this AVM and are removed with the AVM (because the AVM has essentially taken up the space of bilateral tonsils and is sitting in the fourth ventricle) is now the roof of the fourth ventricle has been removed. We can see the entire fourth ventricle, facial colliculi [yellow arrow at 6:05], and then all the way up to the cerebral aqueduct [yellow arrow at 6:08]. So now, we are seeing the entirety of the fourth ventricle after the roof has been removed, which harbored this AVM. We can see the bone that is remaining over the inion [yellow arrow at 6:16] here—this is superior—and so we really get a nice view of the fourth ventricle.

**ng.6:22 Postoperative Imagi We know that this AVM has been removed.** There is no breakthrough bleeding. The vein has been taken at the top of the exposure. We did not perform an angiogram given this patient was pregnant, and we had postoperative CTA, which we will see here (6:33), which shows resection of this AVM. The patient did have a delayed angiogram at a later time.

**6:40 Clinical Course.** She had a prolonged postoperative course and remained in the hospital with monitoring of fetal heart tones, which remained excellent. She was in the hospital for many weeks but eventually was discharged to rehab, where she continued to recover. She had poor PO intake at first, but she did not get a PEG tube and eventually regained the ability to take PO intake. Her tracheostomy had been removed prior to delivery. She delivered a healthy baby boy approximately 4.5 months later, and at 6 months’ follow-up, she was essentially neurologically intact, with some gait ataxia but otherwise walking independently. She has since made a full recovery.

**7:15 Conclusion.** So, this case illustrates a posterior fossa AVM in a pregnant patient who underwent two stages. The first stage was a decompression, followed by the second stage after she showed some improvement following the decompression. The second stage was resection of the AVM and the tonsil, and she ultimately went on, 4.5 months after the surgery, to have a healthy baby boy through a normal vaginal delivery and has since made a full recovery. Thank you.

## Author Contributions

Primary surgeon: Abla. Assistant surgeon: Raper, Raygor, Rutledge. Editing and drafting the video and abstract: Abla, Raper, Raygor, Dubnicoff. Critically revising the work: Abla, Raper, Raygor, Rutledge. Reviewed submitted version of the work: Abla, Raper, Raygor, Rutledge. Approved the final version of the work on behalf of all authors: Abla. Supervision: Abla.
